# Development and comprehensive characterization of porcine hepatocellular carcinoma for translational liver cancer investigation

**DOI:** 10.18632/oncotarget.27647

**Published:** 2020-07-14

**Authors:** Ron C. Gaba, Lobna Elkhadragy, F. Edward Boas, Sulalita Chaki, Hanna H. Chen, Mohammed El-Kebir, Kelly D. Garcia, Eileena F. Giurini, Grace Guzman, Francesca V. LoBianco, Mario F. Neto, Jordan L. Newson, Aisha Qazi, Maureen Regan, Lauretta A. Rund, Regina M. Schwind, Matthew C. Stewart, Faith M. Thomas, Herbert E. Whiteley, Jiaqi Wu, Lawrence B. Schook, Kyle M. Schachtschneider

**Affiliations:** ^1^Department of Radiology, University of Illinois at Chicago, Chicago, IL, USA; ^2^Department of Pathology, University of Illinois at Chicago, Chicago, IL, USA; ^3^Department of Radiology, Memorial Sloan Kettering Cancer Center, New York City, NY, USA; ^4^Department of Animal Sciences, University of Illinois at Urbana-Champaign, Urbana, IL, USA; ^5^Department of Computer Sciences, University of Illinois at Urbana-Champaign, Urbana, IL, USA; ^6^Biological Resources Laboratory, University of Illinois at Chicago, Chicago, IL, USA; ^7^College of Medicine, University of Arkansas for Medical Sciences, Little Rock, AR, USA; ^8^Department of Biochemistry and Molecular Genetics, University of Illinois at Chicago, Chicago, IL, USA; ^9^College of Veterinary Medicine, University of Illinois at Urbana-Champaign, Urbana, IL, USA; ^10^National Center for Supercomputing Applications, University of Illinois at Urbana-Champaign, Urbana, IL, USA

**Keywords:** liver cancer, transgenic pigs, large animal model, interventional radiology, personalized medicine

## Abstract

Hepatocellular carcinoma (HCC) is the second leading cause of cancer-related death worldwide. New animal models that faithfully recapitulate human HCC phenotypes are required to address unmet clinical needs and advance standard-of-care therapeutics. This study utilized the Oncopig Cancer Model to develop a translational porcine HCC model which can serve as a bridge between murine studies and human clinical practice. Reliable development of Oncopig HCC cell lines was demonstrated through hepatocyte isolation and Cre recombinase exposure across 15 Oncopigs. Oncopig and human HCC cell lines displayed similar cell cycle lengths, alpha-fetoprotein production, arginase-1 staining, chemosusceptibility, and drug metabolizing enzyme expression. The ability of Oncopig HCC cells to consistently produce tumors *in vivo* was confirmed via subcutaneous (SQ) injection into immunodeficient mice and Oncopigs. Reproducible development of intrahepatic tumors in an alcohol-induced fibrotic microenvironment was achieved via engraftment of SQ tumors into fibrotic Oncopig livers. Whole-genome sequencing demontrated intrahepatic tumor tissue resembled human HCC at the genomic level. Finally, Oncopig HCC cells are amenable to gene editing for development of personalized HCC tumors. This study provides a novel, clinically-relevant porcine HCC model which holds great promise for improving HCC outcomes through testing of novel therapeutic approaches to accelerate and enhance clinical trials.

## INTRODUCTION

Hepatocellular carcinoma (HCC)—the most common type of primary liver cancer—is an aggressive cancer that spans more than 850,000 new yearly diagnoses and causes 800,000 annual deaths, representing the fifth most common cancer globally and the second most common cause of cancer-related death worldwide [1]. The incidence of HCC in the United States has tripled over the past three decades, and is projected to increase for the foreseeable future given the growing prevalence of HCC risk factors, including hepatitis B or C virus infection, obesity, diabetes mellitus, and excessive alcohol consumption [2]. HCC results from chronic liver disease, termed cirrhosis, with cancer developing at a 5-year incidence up to 30% in at-risk cirrhotic populations [3]. The prognosis for HCC patients is dismal, with an overall 5-year survival rate of 18.4% [4], and the increasing prevalence of liver cirrhosis ensures that HCC will continue to represent an important public health concern in the future [5].

A large number of rodent HCC models have been developed and utilized for preclinical research [6]. Despite their benefits, current HCC animal models have significant disadvantages that limit the testing of novel therapies and their translation to clinical practice. First, rodents are poor preclinical models of drug toxicity, sensitivity, and efficacy due to significant differences in xenobiotic receptors and drug metabolism [7]. This factor is of immense importance as less than 8% of drugs translate successfully from animal testing into Phase 1 clinical cancer trials [8]. Furthermore, the small size of rodents prohibits the testing of device-based tools and techniques widely employed in clinical practice. This is of significant consequence given the central role of locoregional therapies (LRTs) in HCC clinical management. The rabbit VX2 model has been considered the most relevant and widely used model to test HCC LRTs to date [9]. However, the VX2 model also has significant drawbacks, such as squamous cell origin, unknown tumor biology, internal necrosis, only peripheral vascularization, and varying tumor kinetics [10]. As such, there is a crucial need for more clinically relevant large animal models that faithfully recapitulates human HCC to address unmet clinical needs and serve as a bridge between murine studies and clinical practice.

This study describes utilization of the Oncopig Cancer Model for development of a clinically relevant, translational porcine HCC model. The Oncopig Cancer Model is a transgenic pig model that develops site and cell specific tumors following Cre recombinase induced expression of heterozygous *KRAS^G12D^* and *TP53^R167H^* transgenes [11]. The large size of the pig and its similarities with humans in terms of anatomy, physiology, metabolism, immunity, and genetics make it an ideal model species for development of a large animal cancer model. Development of Oncopig HCC cell lines has been previously described [12], however, prior work was limited to characterization of HCC cell lines derived from three Oncopigs, minimal *in vitro* and *in vivo* profiling, and no description of intrahepatic tumors. As such, this study was undertaken to test the hypothesis that phenotypically consistent Oncopig HCC cells that faithfully recapitulate the *in vitro* features of human HCC can be developed across a large Oncopig cohort, and that these cells can be utilized to develop clinically relevant intrahepatic HCC tumors in Oncopigs.

## RESULTS

### Oncopig HCC cells recapitulate *in vitro* features of human HCC cells

**Figure 1:**
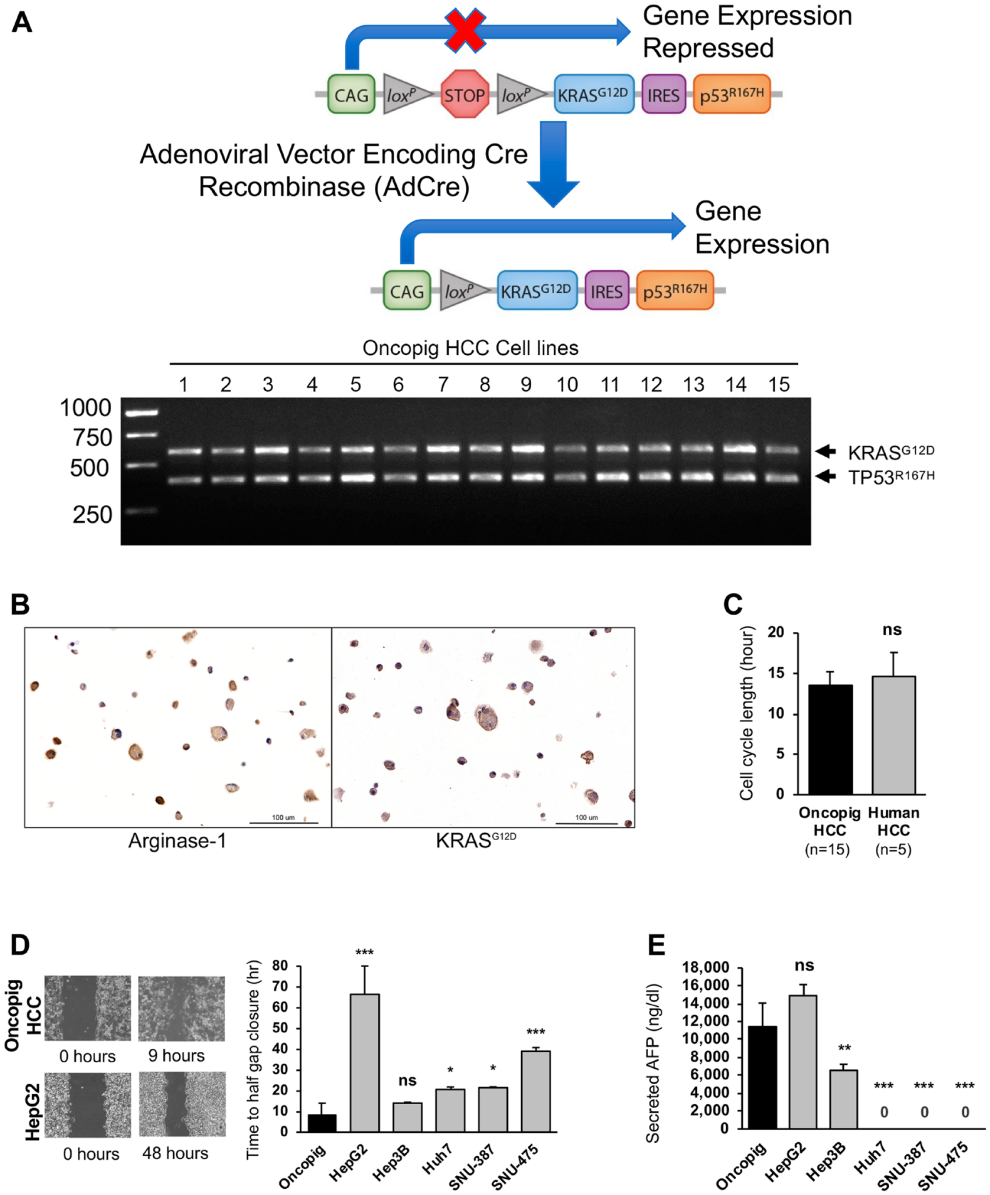
Oncopig and human HCC *in vitro* phenotypes. (**A**) Schematic of Oncopig transgene construct and agarose gel electrophoresis of RT-PCR products confirming Oncopig transgene (*KRAS^G12D^* and *TP53^R167H^*) expression following exposure to AdCre. (**B**) Positive arginase-1 and KRAS^G12D^ staining (brown) of cultured Oncopig HCC cell lines (20×). (**C**) Oncopig and human HCC cell cycle lengths. (**D**) Representative cell migration images depicting faster gap closure in Oncopig compared to HepG2 and half gap closure rates for Oncopig (*n* = 15 cell lines) and human HCC cells. (**E**) AFP secretion from Oncopig (*n* = 15 cell lines) and human HCC cells. Huh7, SNU-387, and SNU475 are known non-AFP producing cell lines. ns = non-significant, ^*^denotes *p*-value < 0.05, ^**^denotes *p*-value ≤ 0.01, ^***^denotes *p*-value ≤ 0.0001.

HCC cell lines were successfully developed from 15 Oncopigs by exposure of isolated hepatocytes to Cre recombinase. As we have previously demonstrated Oncopig primary hepatocytes do not express Oncopig transgenes and become apoptotic within 14 days of culturing [12], primary hepatocytes were not included in any downstream analyses. Characterization of Oncopig HCC cells confirmed expression of *KRAS^G12D^* and *TP53^R167H^*, sustained propagation *in vitro*, positive arginase-1 staining (median 100%, range 88–100% purity), and positive KRAS^G12D^ staining (Figure 1A, 1B). Oncopig HCC cell lines displayed cell cycle lengths that mirrored human HCC cell lines (HepG2, Hep3B, Huh7, SNU-387, and SNU-475; Figure 1C and Supplementary Figure 1). In a wound healing assay, Oncopig HCC cell lines exhibited comparable time to half gap closure as Hep3B cell line (Figure 1D). Oncopig HCC cell lines produced alpha fetoprotein (AFP) at similar levels as HepG2 cells (Figure 1E), demonstrating the potential utility of serum AFP as a biomarker for tumor growth in the Oncopig HCC model, similar to clinical practice [13]. Together, these data demonstrate the generation of 15 distinct Oncopig HCC cell lines displaying consistent *in vitro* features similar to human HCC cells.

### Oncopig HCC is predictive of human HCC chemotherapeutic susceptibility

**Figure 2:**
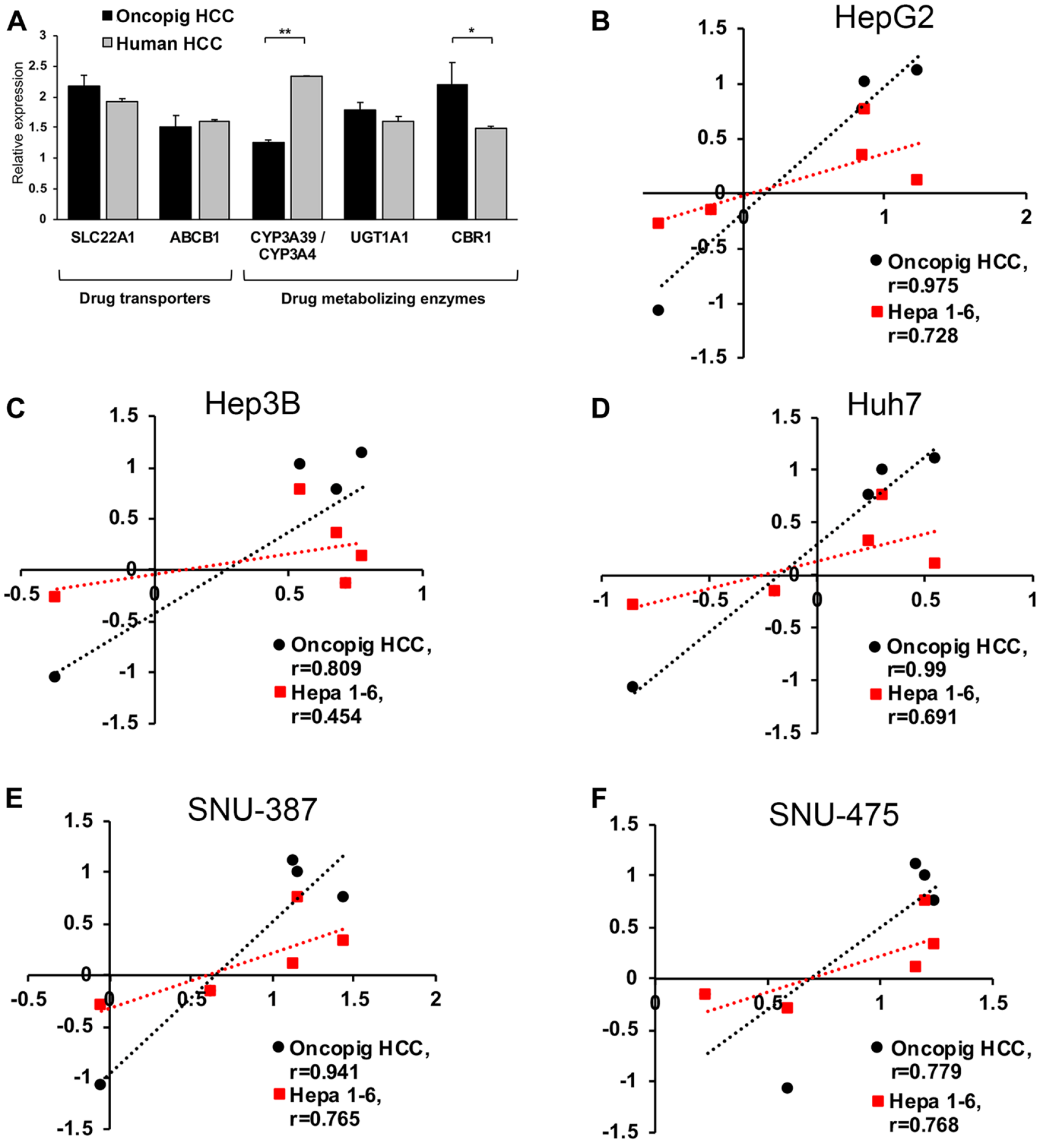
Oncopig, human, and murine HCC *in vitro* chemotherapeutic susceptibility. (**A**) Gene expression levels in Oncopig (*n* = 3 cell lines) and human HCC cells (HepG2, Huh7, and Hep3B). (**B**–**F**) Correlation analysis of logIC_50_ values demonstrating more similar *in vitro* chemotherapeutic responses between Oncopig and human compared to murine Hepa1-6 and human HCC cells. Chemotherapeutic response of each HCC cell line towards sorafenib, doxorubicin, cisplatin, mitomycin C, and 5-FU was determined. Pearson correlation between logIC_50_ in Oncopig HCC cells or murine Hepa1-6 cells and the following human HCC cells was analyzed: (B) HepG2, (C) Hep3B, (D) Huh7, (E) SNU-387, and (F) SNU-475. ^*^denotes *P* < 0.05, ^**^denotes *P* ≤ 0.001.

As comparative expression of genes involved in drug metabolism and transport between animal models and humans can predict similarities in treatment responses [14], expression levels of key drug metabolizing enzymes and transporters were compared in human (HepG2, Huh7, and Hep3B) and Oncopig HCC cells. Similar expression of the uptake transporter *SLC22A1*, the efflux pump *ABCB1*, as well as the drug metabolizing enzyme *UGT1A1* [15–17], were observed between Oncopig and human HCC cell lines (Figure 2A). In contrast, reduced expression of the phase 1 sorafenib metabolizing enzyme *CYP3A4* (porcine homologue *CYP3A39*) [18, 19] and increased expression of *CRB1*, which is involved in doxorubicin metabolism [20] were observed in Oncopig cell lines compared to human HCC cell lines (Figure 2A).

To evaluate the ability of the Oncopig HCC model to predict human HCC therapeutic responses, Oncopig, human, and murine HCC cell line chemotherapeutic susceptibility was tested for agents clinically employed for locoregional (doxorubicin, cisplatin, and mitomycin C) and systemic (sorafenib) HCC treatment [21–23], in addition to 5-fluorouracil (5-FU), which is relatively ineffective for HCC treatment [24]. Oncopig HCC cell lines (*n* = 6) displayed consistent susceptibility to both sorafenib and doxorubicin (Supplementary Figure 2A, 2B). In addition, Oncopig HCC cell line log half maximal inhibitory concentrations (logIC_50_) values were highly correlated with logIC_50_ values for all five human HCC cell lines (Pearson’s r = 0.779–0.990; Figure 2B–2F and Supplementary Table 1), suggesting Oncopig HCC chemotherapeutic responses are highly predictive of human responses. In addition, Oncopig HCC responses were more predictive than the murine HCC line Hepa1-6, as evidenced by higher correlation coefficients for Oncopig compared to murine HCC cells for all 5 human comparisons (Figure 2B–2F). Importantly, while Oncopig HCC cells correctly predicted resistance of human HCC to 5-FU treatment, murine HCC cells displayed markedly increased susceptibility to 5-FU (Supplementary Figure 2C). Together, these results demonstrate similar expression of key genes involved in drug metabolism and transport between Oncopig and human HCC, and the ability of Oncopig HCC cells to predict human HCC chemotherapeutic susceptibility *in vitro*, providing a rationale for utilization of the Oncopig HCC model as a translational model to bridge the gap between murine and human studies.

### Oncopig HCC cells produce viable SCID mouse xenografts

**Figure 3:**
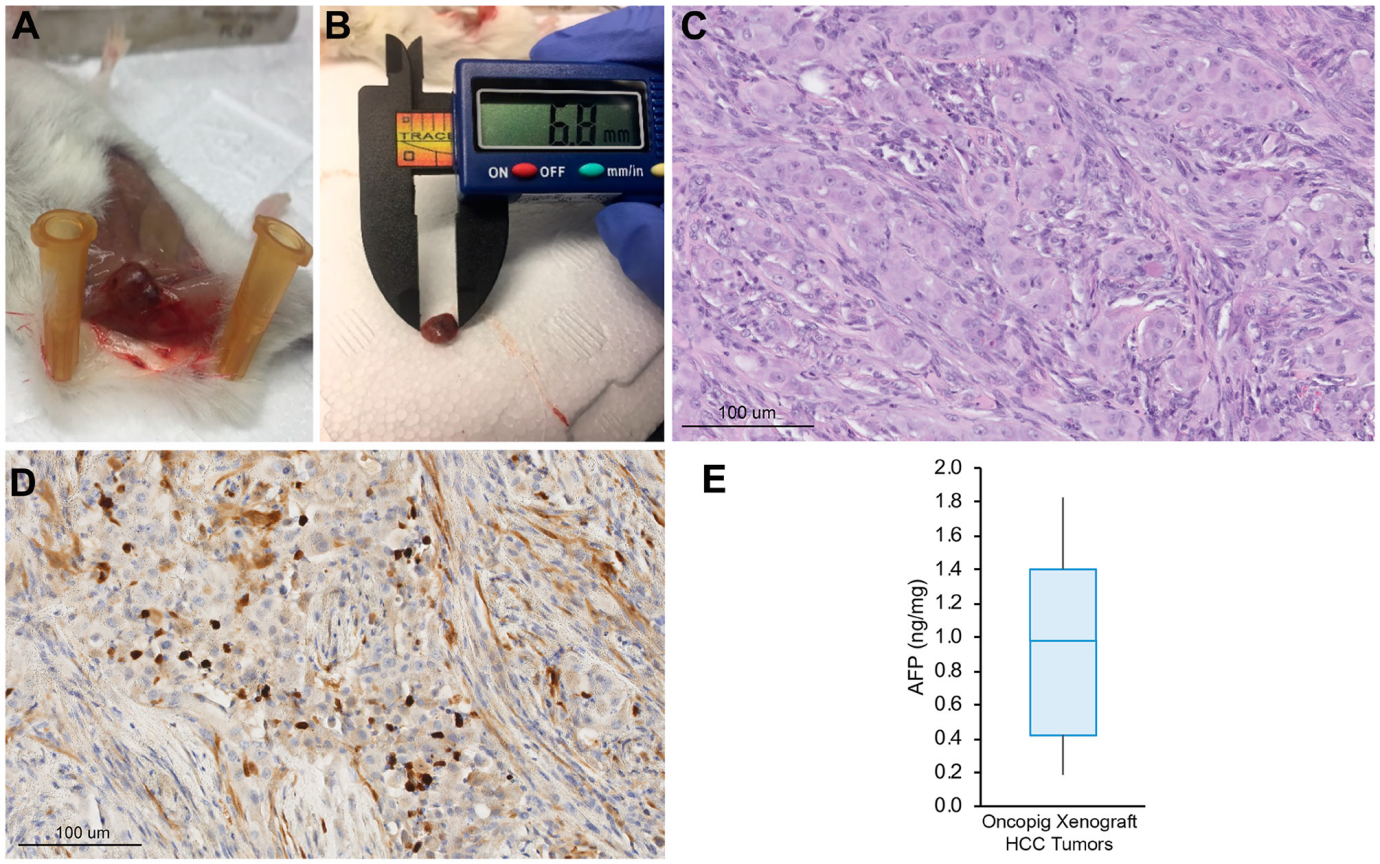
Oncopig HCC xenograft tumor development. (**A**) Representative SQ Oncopig HCC xenograft tumor. (**B**) Excised Oncopig HCC xenograft tumor. (**C**) H & E (20×) of Oncopig HCC xenograft tumor reveals densely cellular subcutaneous nodule with interspersed fat cells. Intervening fibrous vascular septae noted. (**D**) On arginase-1 IHC (20×), epithelial cells show focal arginase-1 expression (brown) consistent with hepatocellular differentiation. (**E**) AFP expression across Oncopig HCC xenograft tumors (*n* = 10).

To confirm *in vivo* tumorigenicity, Oncopig HCC cell lines (*n* = 15) were injected subcutaneously (SQ) into severe combined immunodeficiency (SCID) mice. In total, 68 SQ Oncopig HCC tumors were successfully developed (median 4, range 3–8 tumors/cell line; Figure 3A, 3B). Tumors measured median 6.7 × 5.4 mm in size at 21-days post injection. Histologic evaluation confirmed neoplastic masses comprised of spindle shaped malignant cells displaying arginase-1 positivity (Figure 3C, 3D). In addition, whereas control untransformed Oncopig tissues did not produce AFP, Oncopig xenograft HCC tumors produced AFP (Figure 3E), further confirming their identity as HCC tumors.

### Oncopig HCC cell lines reproducibly generate autograft SQ tumors

**Figure 4:**
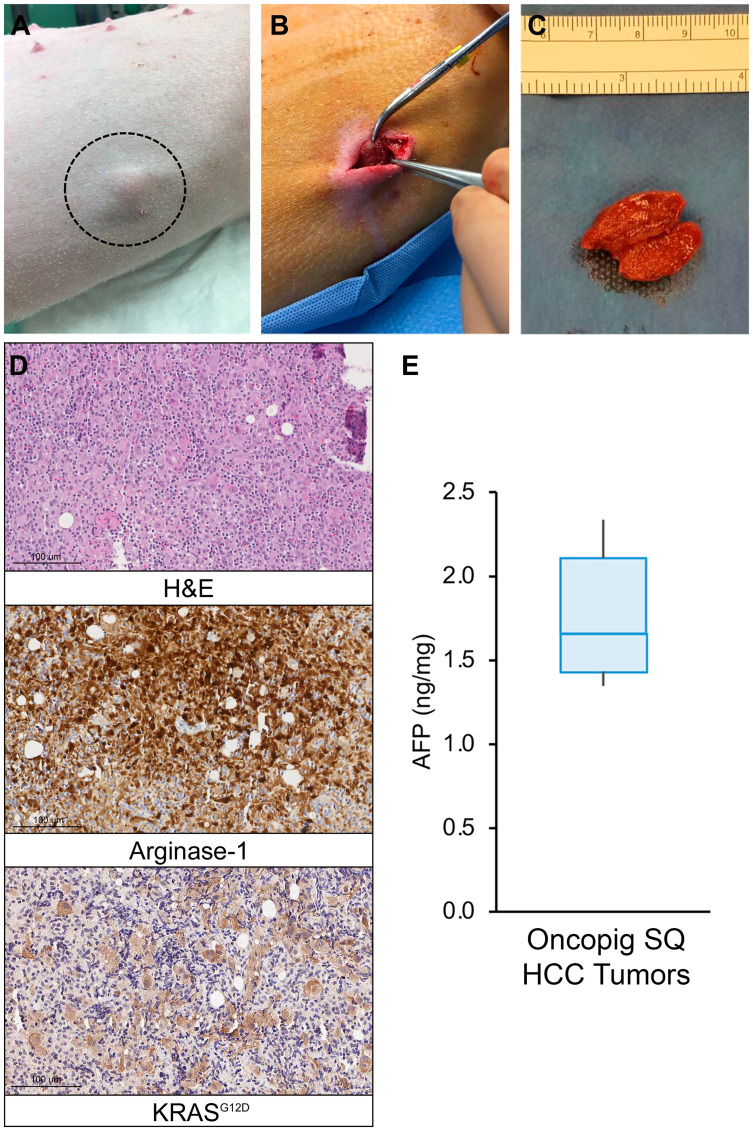
Oncopig SQ HCC autograft formation. (**A**) Photograph of visible SQ HCC tumor (circled) in Oncopig flank. (**B**) Excision of 2.0 cm SQ HCC tumor. (**C**) Excised and transected SQ HCC tumor. (**D**) H & E (20×) of Oncopig SQ HCC tumor demonstrates prominent, dispersed, pleomorphic large atypical cells, 5–10× the size of lymphocytes, flanking fibrous vascular septae, surrounded by dense mixed immune cell infiltrates. Arginase-1 IHC (20×) shows that these atypical cells show patchy arginase-1 expression (brown) consistent with hepatocellular differentiation. KRAS^G12D^ IHC (20×) confirms KRAS^G12D^ expression (brown) consistent with malignancy. (**E**) AFP expression across Oncopig SQ HCC tumors (*n* = 6).

In order to confirm the reproducibility of our previous report demonstrating development of a single SQ Oncopig HCC tumor [12], autologous SQ injection of Oncopig HCC cells was performed across 13 Oncopigs, which resulted in successful tumor formation at a rate of 68% (34/50) per injection and 85% (11/13) per pig (Figure 4A–4C), reaching a median size of 16 × 13 mm within 2-weeks post injection. Histologic evaluation confirmed neoplasm in all cases, characterized by malignant epithelial cells with invasion into adjacent skeletal muscle, regions of inflammation, and positive arginase-1 and KRAS^G12D^ staining (Figure 4D). Oncopig SQ HCC tumors also produced AFP (Figure 4E), further confirming their identity as HCC tumors. Control Oncopig tissues were negative for AFP production.

### Reproducible development of Oncopig intrahepatic HCC tumors

**Figure 5:**
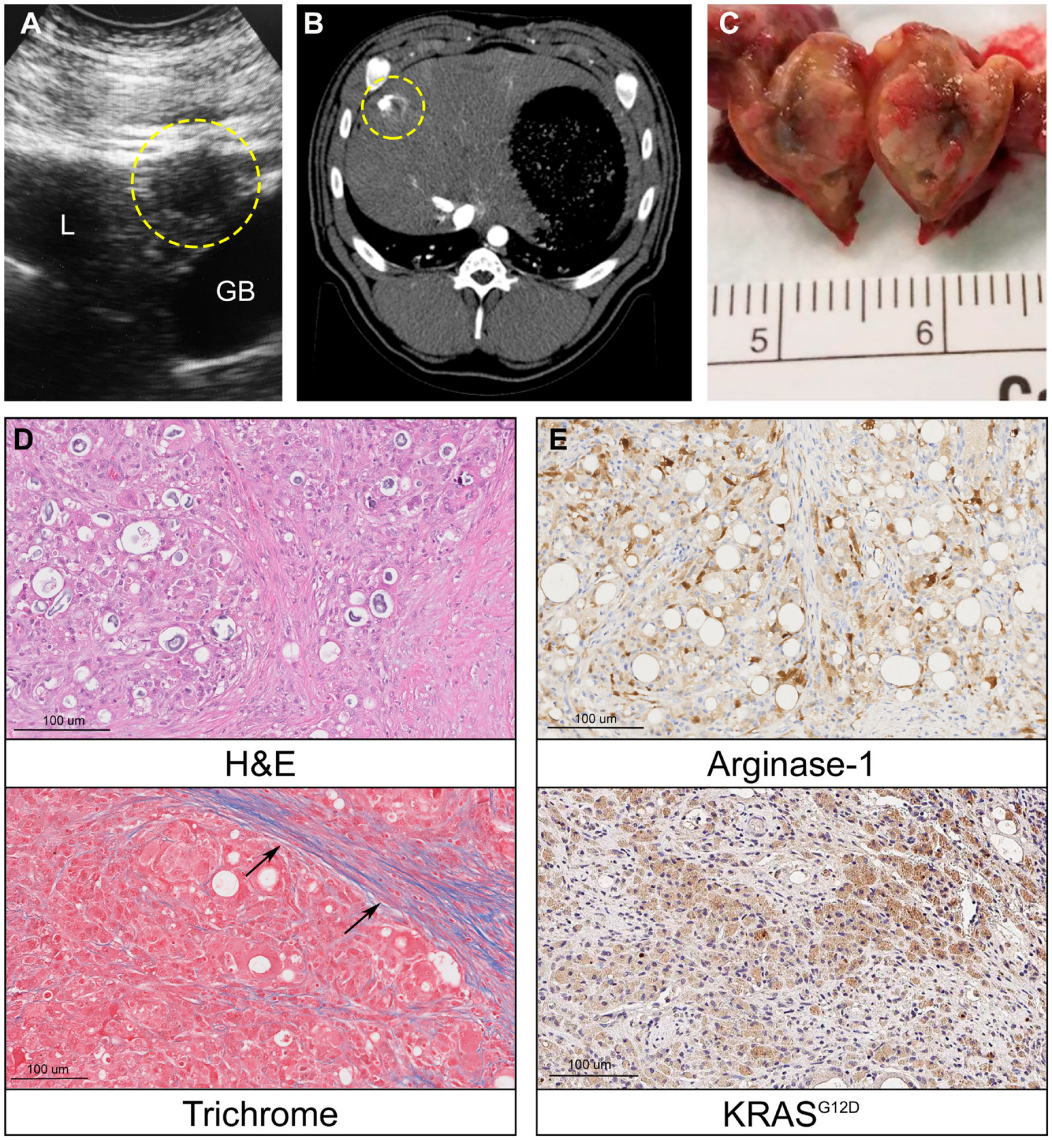
Oncopig intrahepatic HCC tumor formation. (**A**) Liver ultrasound depicting a hypoechoic 1 cm round intrahepatic HCC tumor (circled, L = liver, GB = gallbladder). (**B**) Contrast enhanced liver CT depicts same HCC tumor (circled). (**C**) Photograph of transected intrahepatic HCC tumor. (**D**) H & E (20×) of Oncopig intrahepatic HCC tumor reveals architectural distortion characterized by expansion of liver cords, nuclear pleomorphism, anisonucleosis, and nodular fibrosis. Masson’s trichrome of adjacent non-tumorous liver demonstrates dense collagen bands (arrows) consistent with METAVIR grade 2-3 fibrosis. (**E**) Arginase-1 IHC (20×) shows patchy arginase-1 expression (brown) consistent with hepatocellular differentiation. KRAS^G12D^ IHC (20×) confirms KRAS^G12D^ expression (brown) consistent with malignancy.

Following confirmation of successful, reproducible Oncopig SQ HCC tumor formation, Oncopig intrahepatic HCC tumor development was performed via autologous engraftment of SQ tumor fragments into Oncopig livers following 15 days of SQ tumor growth. Consistent with findings in murine HCC models demonstrating improved tumor growth when implanting HCC cells into cirrhotic compared to non-cirrhotic livers [25], attempts to develop intrahepatic tumors in healthy Oncopig livers were unsuccessful (*n* = 3). Therefore, in order to allow for development in a clinically relevant liver microenvironment, alcoholic liver fibrosis was induced immediately prior to engraftment in a single Oncopig. The Oncopig underwent biweekly ultrasound surveillance for intrahepatic tumor formation, which resulted in identification of a 1.0 cm mass 4-weeks post engraftment (Figure 5A). Intrahepatic HCC tumor formation was confirmed 5 days later via computed tomography (CT) scan (Figure 5B). The Oncopig was then euthanized and tumor samples were collected for histological and genomic analyses (Figure 5C). Histological evaluation confirmed an HCC tumor showing architectural distortion characterized by expansion of liver cords, nuclear pleomorphism, anisonucleosis, and nodular fibrosis in a background of dense collagen bands within adjacent non-tumorous liver consistent with METAVIR grade 2–3 fibrosis (Figure 5D), as well as arginase-1 and KRAS^G12D^ positivity (Figure 5E).

Following confirmation of successful Oncopig intrahepatic HCC tumor formation, the ability to reproducibly develop Oncopig intrahepatic HCC tumors was confirmed by performing HCC cell line development, SQ injection, and concurrent liver fibrosis induction and engraftment of SQ tumor fragments into the liver of 2 additional Oncopigs following 11 days of SQ tumor growth. Consistent with the first experiment, imaging assessment with ultrasound and CT resulted in identification of liver tumors. These included a hypoechoic intrahepatic mass measuring 1.0 cm 4-weeks post engraftment in the first Oncopig (Supplementary Figure 3A) that was not visible on subsequent follow-up, and a hypoechoic liver mass measuring 0.6 cm 2-weeks post engraftment that grew to 1.4 cm 4-weeks post engraftment in the second Oncopig (Supplementary Figure 3B, 3C). Finally, a hypervascular liver tumor measuring 1.0 cm was observed 10-weeks post tumor engraftment in the second Oncopig (Supplementary Figure 3D). Together, these results demonstrate reproducible development of Oncopig intrahepatic HCC tumors of clinically relevant sizes, in addition to the ability to image and characterize using clinically relevant modalities.

### Genomic signatures of Oncopig intrahepatic HCC

**Figure 6:**
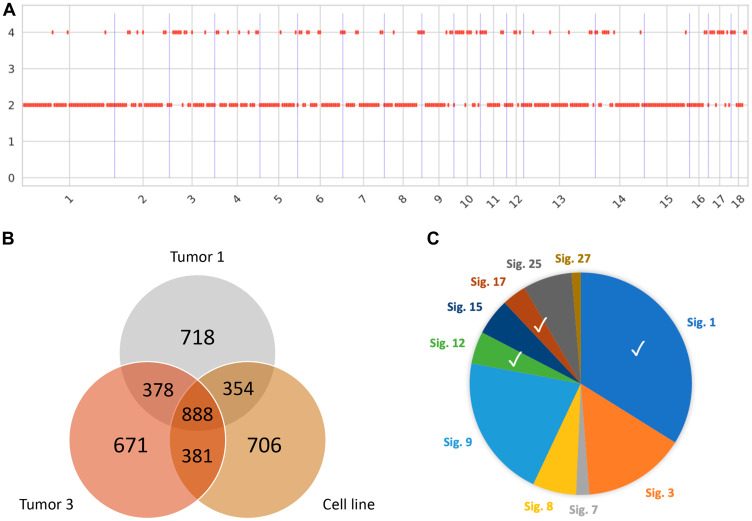
Genomic signatures of Oncopig HCC. (**A**) Somatic copy-number calling reveals a largely copy-neutral tumor in line with the young age of the tumor. (**B**) Representative venn diagram showing distribution of SNVs in the cell line and 2 out of 5 tumor samples. (**C**) Mutational signatures identified resemble signatures observed in human HCC tumors (Signatures 1, 12, and 17).

In order to assess the ability of Oncopig HCC to mimic human HCC genomic signatures observed clinically, five spatially distinct tumor biopsies from the intrahepatic HCC tumor depicted in Figure 5C underwent whole-genome sequencing. Consistent with the relatively young age of the tumor (5 weeks old), copy number calling showed a mostly copy-neutral tumor (Figure 6A). In total 5,257 single nucleotide variants (SNVs) were identified, with 337 variants common between each tumor sample, and between 249 to 385 SNVs unique to each sample (Figure 6B and Supplementary Figure 4), indicating the presence of intratumor heterogeneity resulting from the accumulation of somatic mutations in distinct tumor cells as commonly observed in human HCC. To assess whether the SNVs resulted from mutational processes observed in human HCC, Oncopig mutational profiles were decomposed into mutational signatures, resulting in identification of 11 COSMIC v2 signatures (Figure 6C). Of these, signature 1 (33.7%) is observed in almost all human tumors, while signatures 12 and 17 (4.7% and 3.5%, respectively) are associated with human HCC. Finally, 133 variants were found to occur in 89 driver genes, all of which are known to be mutated in human HCC (Supplementary Table 2). Although these variants did not occur in coding regions, studies have suggested that noncoding mutations can affect gene regulation and may be important to elucidate mechanisms of tumorigenesis [26]. In summary, intratumor heterogeneity and SNV signatures previously identified in human HCC were observed in Oncopig intrahepatic HCC, indicating Oncopig HCC tumors resemble human HCC at the genomic level.

### Oncopig HCC cell lines are genetically manipulatable

**Figure 7:**
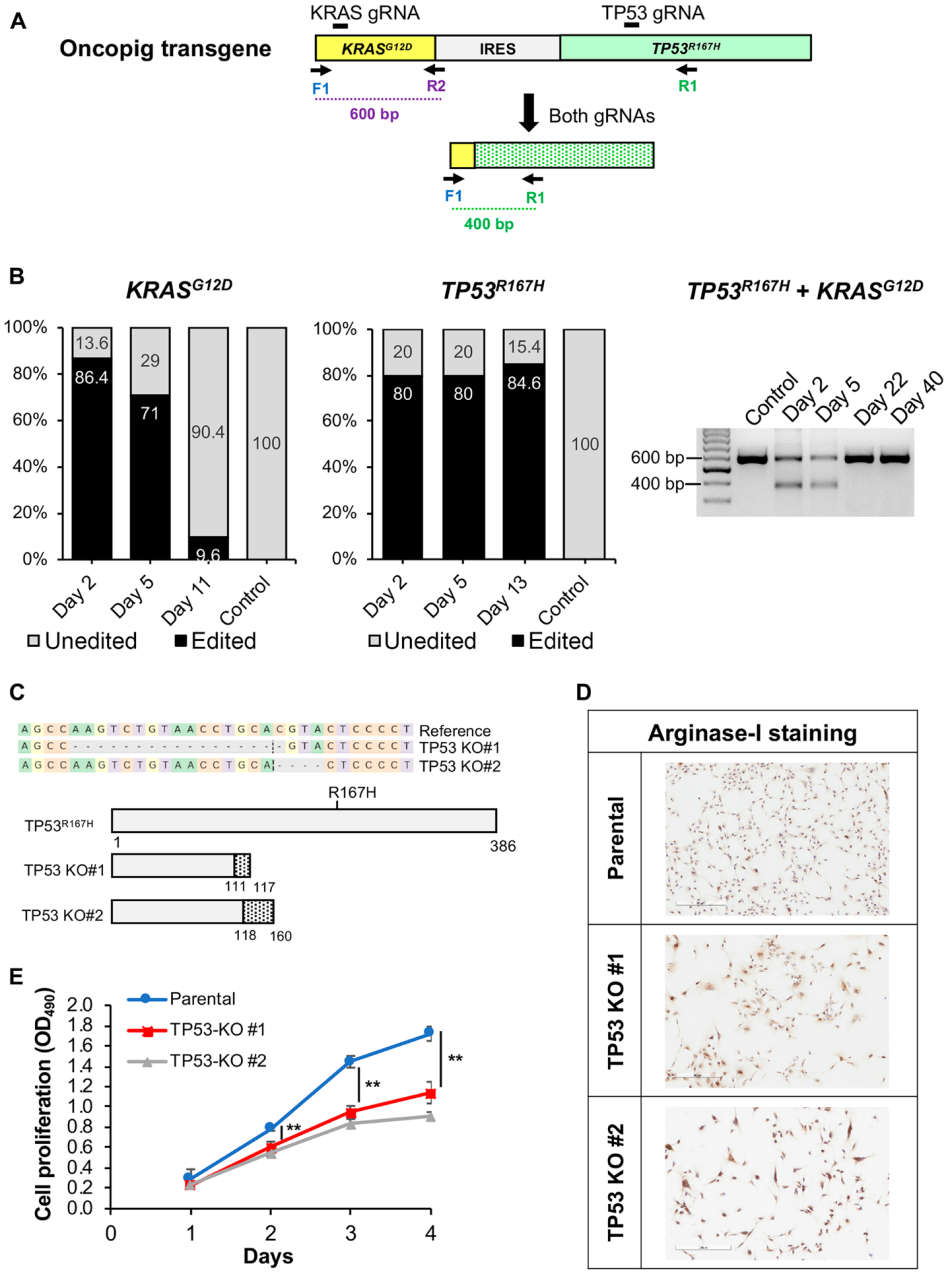
CRISPR/Cas9-mediated disruption of Oncopig KRAS^G12D^ and TP53^R167H^ transgenes. (**A**) Schematic representation of the Oncopig transgene showing gRNA target sites and primers used for PCR. IRES, Internal ribosome entry site. (**B**) *KRAS^G12D^* and *TP53^R167H^* editing efficiencies at multiple time points post transfection with Cas9 and gRNAs. (**C**) Frameshift mutations resulting in protein truncation for 2 Oncopig *TP53^R167H^* KO HCC cell lines developed via single cell clone isolation and screening. Dashed line marks the cleavage position, and dashed grey boxes represent nucleotide deletions. Dotted regions represent frameshifts in predicted protein sequences. (**D**) Positive arginase-1 staining (brown) of parental and *TP53^R167H^* KO cell lines (scale bar, 300 μm). (**E**) Cellular proliferation of Oncopig parental and *TP53^R167H^* KO HCC cell lines. Values represent mean ± S. D. (*n* ≥ 3). ^**^indicates *P* < 0.001.

Advances in animal modeling and gene editing provide an opportunity to develop genetically tailored tumors. This enables investigation of the contribution of clinically relevant driver mutations on tumor progression and treatment susceptibility, as well as preclinical testing of novel precision medicine approaches. As a first step towards generation of genetically tailored Oncopig HCC tumors, we tested our ability to knockout (KO) the Oncopig *TP53^R167H^* and *KRAS^G12D^* driver mutations using CRISPR-Cas9 (Figure 7A). Oncopig HCC cells were successfully edited at a rate of over 80% (Figure 7B) with insertions or deletions (INDELs) occurring around the predicted cleavage sites (Supplementary Figure 5A, 5B). In addition, simultaneous targeting of *TP53^R167H^* and *KRAS^G12D^* resulted in deletion of the region between the two gRNAs (Figure 7A, 7B) which was confirmed via Sanger sequencing (Supplementary Figure 5C). While the percentage of *TP53^R167H^* edited cells was maintained in culture for up to two weeks, the proportion of cells harboring *KRAS^G12D^* edits decreased over time (Figure 7B) suggesting *KRAS^G12D^* is required for Oncopig HCC cell survival. Isolation and screening of 5 single cell clones from the *TP53^R167H^* edited cell pool resulted in development of two *TP53^R167H^* KO HCC cell lines harboring frameshift mutations (17 and 4 bp deletions) leading to protein truncation (Figure 7C). The parental and *TP53^R167H^* KO cell lines stained positive for arginase-1 (Figure 7D), confirming their identity as HCC cells. As expected, *TP53^R167H^* KO resulted in reduced cell proliferation compared to the parental line (Figure 7E) further demonstrating the ability to introduce genetic alterations with significant effects on malignant potential. These results suggest combining the Oncopig orthotopic HCC model with *in vitro* gene editing could lead to development of genetically tailored HCC tumors for investigating the contribution of driver mutations on clinically relevant cancer phenotypes and testing of novel precision medicine approaches.

## DISCUSSION

Advances in cancer care are dependent upon the use of preclinical *in vivo* model systems to test new diagnostic and imaging modalities, therapeutic strategies, and improve treatment outcomes. Due to the limitations of current HCC animal models, more clinically relevant large animal HCC models are required to more effectively test novel LRTs and other therapies for translation to clinical practice. This study presents a genetically adjustable, reproducible Oncopig HCC model that closely predicts human HCC chemotherapeutic responses. Expanding on previous reports [12], reproducible generation of Oncopig SQ HCC tumors via autologous injection of Oncopig HCC cells is demonstrated, in addition to engraftment of SQ tumor fragments into the liver resulting in reproducible development of intrahepatic HCC tumors that reach clinically relevant sizes within 1–2 months and are trackable using clinically employed imaging modalities. Although alcohol-induced liver fibrosis was observed in liver adjacent to intrahepatic tumors, it did not reach an irreversible cirrhotic state (METAVIR grade 4), indicating further work is required to increase the severity of liver disease in this model. This result is further confirmed by our previous publication demonstrating alcohol-induced liver fibrosis disease severity peaks at 8-weeks post induction, with fibrosis levels reducing to METAVIR grade F1–F2 by 20-weeks post induction [27]. Liver fibrosis recovery during tumor development could also explain the lack of sustained intrahepatic tumor growth observed over the 10-week observation period, although further studies in a larger cohort are required to confirm this hypothesis.

The successful development and implementation of novel therapeutic strategies for HCC is critically dependent on the capability to screen, test, and apply such approaches across the *in vitro* to *in vivo* continuum. The presented *in vitro* chemotherapy results support the concept that the Oncopig HCC model can be used to screen and test promising drugs with results translatable to clinical trials, and subsequently clinical practice. The Oncopig HCC model therefore represents an ideal platform that permits *in vitro* therapeutic screening using HCC cell lines with the ability to translate promising strategies to *in vivo* testing in a clinically relevant large animal model. In addition, as pigs are commonly used for toxicology studies [28], the Oncopig HCC model provides the opportunity to simultaneously test safety and efficacy in the same animal. However, as *in vivo* chemotherapeutic responses were not assessed in this study, it is unclear whether Oncopig HCC *in vitro* chemotherapeutic responses are representative of *in vivo* responses. This represents a limitation of the current study, highlighting the need for future studies assessing Oncopig intrahepatic HCC tumor chemotherapeutic susceptibility.

While the Oncopig HCC model is not the only porcine HCC model reported to date, it does provide significant advantages over previously published models. Autochthonous HCC has been developed in chemically induced porcine models [29–31]; however, such models take 1–2 years to develop clinically relevant tumors and do not allow for control of tumor number, location, underlying genetics, or comorbidities, rendering them potentially less suitable for preclinical and co-clinical trials. On the other hand, the Oncopig HCC model presented allows for development of genetically defined intrahepatic HCC tumors of clinically relevant size in animals as young as 4–5 months of age. In addition, Oncopig HCC tumors recapitulate human histologic and molecular features, including arginase-1 staining and AFP production. Another potentially significant advantage of the Oncopig HCC model is highlighted by our successful development of genetically defined Oncopig HCC cell lines. This work provides proof-of-concept validation for combining the Oncopig orthotopic HCC model with *in vitro* gene editing to develop genetically tailored HCC tumors that can be used for investigating the contribution of driver mutations on cancer phenotypes and testing of novel precision medicine approaches. In addition, the large size and segmental nature of the porcine liver enables development of several distinct spatially separated tumors in the same animal. Hence, one animal can be used to investigate therapeutic effectiveness on tumors bearing differential mutational profiles. Further studies are required to assess the ability to develop genetically tailored HCC tumors through knockout of tumor suppressor genes or introduction of activating oncogenic mutations.

In summary, the Oncopig HCC model offers a novel, physiologically and anatomically relevant cancer model for which a multitude of innovative therapeutic modalities can be applied and tested while significantly reducing the costs, confounding variables seen in human subjects, and lengthy conduct of human clinical trials. Importantly, the Oncopig can be utilized to conduct correlative studies for more efficient and consistent investigation of new therapies. Its size allows for utilization of the same methods and instruments used in human clinical practice, including CT and magnetic resonance imaging technologies. This model is thus amenable to developing and establishing medical imaging standards related to diagnosing HCC tumors and tracking treatment response using accepted radiologic criteria, a critical facet of therapeutic discovery and validation. Importantly, the Oncopig is also immunocompetent, lending itself to investigation of immunotherapies [32]. Therefore, the Oncopig fulfills the currently unmet clinical modeling needs for HCC, particularly for pilot investigations of experimental therapies or experimental therapeutic combinations not feasible in human subjects.

## MATERIALS AND METHODS

### Animal subjects

This study was completed at the University of Illinois at Urbana-Champaign (UIUC) and the University of Illinois at Chicago (UIC). Institutional Animal Care and Use Committee approval was obtained at both sites (UIUC: protocol #16-065 approved 6/23/2016, UIC: protocol #16-090 approved 7/13/2016). All animals received humane care according to the criteria outlined in the Guide for the Care and Use of Laboratory Animals. Seventeen female Oncopigs were utilized for this study.

### Oncopig HCC cell line development

Oncopig HCC cell lines were developed as previously described [12]. *KRAS^G12D^* and *TP53^R167H^* expression was confirmed by RT-PCR using primers listed in Supplementary Table 3 as previously described [12].

### Cell culture

Human (HepG2, Hep3B, Huh7, SNU-387, and SNU-475; ATCC, Manassas VA), murine (Hepa1-6; ATCC), and Oncopig HCC cell lines were maintained in DMEM (or DMEM/F12 for HepG2) supplemented with 10% FBS and 1% Penicillin-Streptomycin.

### Proliferation assays

Oncopig and human HCC cell cycle lengths were analyzed using the Cell Trace CFSE Cell Proliferation Kit (Thermo Fisher Scientific, Waltham MA, USA) following the manufacturer’s instructions. Cell cycle length was calculated using linear regression estimation from a percent fluorescence versus time plot. The proliferation of Oncopig HCC *TP53^R167H^* knockout cells was determined using the CellTiter 96 AQueous One Solution Cell Proliferation Assay Kit (#G3580; Promega, Madison WI, USA) following the manufacturer’s instructions.

### Cell migration

Cell migration was analyzed by plating 3 × 10^4^ HCC cells into each chamber of a cell culture insert (Ibidi #81176, Munich, Germany). The cell culture insert was removed 24-hours post seeding, leaving a defined cell-free gap of 500 μm between confluent cell monolayers. Images were taken at 10× magnification at multiple time points until gap closure. Image analysis was performed using the ImageJ [33] MRI Wound Healing Tool plugin (NIH, Rockville MD, USA). Gap area was quantified at each time point, and time to half gap closure was calculated using linear regression estimation from a gap area versus time plot.

### *In vitro* chemotherapeutic susceptibility

The sensitivity of Oncopig, human, and murine HCC cell lines was tested for five chemotherapeutic agents: sorafenib (Bayer, Leverkusen Germany), doxorubicin (Pfizer Inc., New York NY, USA), cisplatin (Sigma-Aldrich, St. Louis MO, USA), mitomycin C (Accord Healthcare Inc., Durham NC, USA), and 5-FU (Acros Organics, Geel Belgium). Briefly, 1 × 10^4^ cells/well (2 × 10^4^ for HepG2) were seeded in 96-well plates. The following day, culture medium was replaced with fresh medium supplemented with each chemotherapeutic agent at 8-point serial dilutions. The following drug concentrations were used to assay clinically relevant administered dosages [34, 35], and to allow calculation of the IC_50_: sorafenib and mitomycin C: 0.5–100 μM, doxorubicin: 0.1–20 μM, cisplatin: 1–200 μM, and 5-FU: 1–500 μM. Cell viability was assessed after 72 hours using a MTT assay (#V13154; Invitrogen, Carlsbad, CA, USA) following the manufacturer’s recommendations using a BioTek 800 TS Absorbance Reader (BioTek, Winooski, VT, USA). IC_50_ values were determined by nonlinear regression analysis using GraphPad Prism 8 (GraphPad, San Diego, CA, USA) from plots of relative percent viability versus log_10_ drug concentration.

### Gene expression analysis

qPCR was performed to assess the expression of genes involved in drug uptake (human: *SLC22A1*; porcine homolog: *SLC22A1*), metabolism (human: *CYP3A4*, *CBR1*, *UGT1A1*; porcine homologs: *CYP3A39*, *CBR1, UGT1A1*) and export (human: *ABCB1*; porcine homolog: *ABCB1*) [15–20]. Total RNA was extracted from Oncopig and human HCC cell lines (HepG2, Huh7, Hep3B) using the All Prep DNA/RNA extraction kit (Qiagen, Germantown MD, USA) according to the manufacturer’s protocol. cDNA was synthesized from RNA using the High-Capacity cDNA Reverse Transcription kit (Thermo Fisher Scientific). qPCR was performed using primers listed in Supplementary Table 3 on the ABI Real Time PCR system (Thermo Fisher Scientific) using the Power SYBR Green Gene PCR Master Mix (Thermo Fisher Scientific). The relative expression level for each gene of interest was determined by normalizing to *GAPDH*.

### Oncopig HCC xenografts

Ten- to 12-week-old female SCID mice (#001303 NOD. CB17-*Prkdc^scid^*/J; Jackson Laboratory, Bar Harbor ME, USA) were used for xenograft tumor generation. Mice were anesthetized by intraperitoneal injection of ketamine (100 mg/kg) and xylazine (5–10 mg/kg). The flanks were shaved and sterilized with alcohol. 1 × 10^7^ Oncopig HCC cells suspended in 50 μL serum free DMEM were SQ injected into each flank using a 21-gauge needle. Mice were sacrificed in a carbon dioxide (CO_2_) chamber followed by cervical dislocation at 21-days post-inoculation. Tumors were harvested by incising the overlying skin, and carefully freeing tumors from SQ tissues using blunt dissection. Tumors were formalin fixed for histologic processing, or homogenized in PBS for AFP analysis.

### Oncopig HCC SQ autografts

Oncopigs were anesthetized by intramuscular administration of telazol (2.6–4.4 mg/kg), ketamine (1.3–2.2 mg/kg), and xylazine (1.3–2.2 mg/kg). The flanks were shaved and sterilized with betadine and alcohol scrub. 1 × 10^7^ Oncopig HCC cells were suspended in 100 μL PBS and autologously injected SQ into 1–6 flank sites per pig (median age 83, range 60–126, days). Oncopigs were euthanized at median 28 (range 21–77) days post SQ tumor inoculation. Tumors were extracted and formalin fixed for histologic processing, or homogenized in PBS for AFP analysis.

### Oncopig intrahepatic HCC tumor development

SQ Oncopig HCC tumor fragments were autografted into the liver following alcohol-induced fibrosis induction in three Oncopigs 11–15 days post SQ injection. Fibrosis was induced by hepatic transarterial infusion of 0.75 mL/kg ethanol-ethiodized oil (1:3 v/v) as described previously [27]. Intrahepatic tumor induction was performed 30 minutes after fibrosis induction. A flank mass was surgically excised, and three 3- to 4-mm^3^ tumor fragments were harvested. Next, the accessible right medial hepatic lobe was identified using ultrasound guidance. A 15-gauge needle with stylet (Echogenic Co-axial Introducer Needle; Argon Medical Devices, Wheeling IL, USA) was advanced percutaneously into the liver, and the Oncopig HCC fragments were pushed into the hepatic parenchyma using the stylet. Ultrasound imaging was performed to monitor for tumor growth and collect tumor biopsies, followed by multiphase contrast-enhanced CT to confirm tumor development (CT protocol: non-contrast, arterial phase at 30–35 seconds, portal venous phase at 60–70 seconds, and delayed phase at 180 seconds after 120–150 mL of iodinated contrast injected intravenously at a rate of 4–5 mL/s). Following CT confirmation, Oncopig subjects were euthanized and tumor masses were harvested, transected, and measured. Tumor samples were either stored in formalin for histologic processing, or flash frozen in liquid nitrogen and stored at –80°C for genomic analysis.

### AFP quantification

AFP levels were determined using a porcine AFP ELISA kit (#MBS945039; MyBioSource Inc., San Diego CA, USA). 3 × 10^6^ HCC cells in 2 ml medium were seeded into 6-well plates and incubated for 72 hours followed by withdrawal of 100 μl of supernatant for analysis. The assay was performed in triplicate for each cell line. For tumor AFP levels, 100 mg was homogenized in 1 ml PBS, followed by 2 freeze-thaw cycles to break the cell membranes. Homogenates were then centrifuged for 5 minutes at 5,000 × g at 4°C and the supernatants were assayed in triplicate immediately following the manufacturer’s protocol. Untransformed Oncopig brain, lung, kidney, and bladder tissues were used as negative controls.

### Immunohistochemistry (IHC)

Oncopig HCC cells in a nearly confluent T75 cm^2^ flask were gently scraped in ice-cold PBS and centrifuged at 100 g at 4°C for 5 minutes. The pellets were re-suspended in 10% formalin and the cells were fixed for 4 hours at room temperature. Cells were then centrifuged at 100× g and the cell pellet was re-suspended in 2% agarose and stored at 4°C for 30 minutes, followed by the addition of 70% ethanol. Cell pellets and formalin fixed tumor samples were provided to the Research Histology and Tissue Imaging Core at the UIC for processing, embedding, sectioning, and staining. Oncopig HCC *TP53*^R167H^ knockout cells were seeded in a chamber slide (#PEZGS0416; Millipore-Sigma, Burlington MA, USA) and incubated overnight. Cells were then washed with PBS, fixed with 10% formalin and provided for staining. Tissue sections were stained using hematoxylin and eosin (H&E). IHC was performed to detect arginase-1 (#ab91279; Abcam; Cambridge, UK) and KRAS^G12D^ (#ab221163; Abcam). Whole slides were scanned using a Hamamatsu Nanozoomer scanner (Hamamatsu Photonics, Hamamatsu City Japan), and digital images were visualized with NDP. view2 software (Hamamatsu Photonics). Cell line purity was quantified by determining the number of arginase-1 positive cells divided by total number of cells across three 40× microscopic regions. Blinded histopathological analyses were performed by a board-certified veterinary pathologist, and board-certified human pathologist with subspecialty training in Liver and Transplantation Pathology.

### CRISPR-Cas9 gene editing

As the Oncopig transgenes are inserted as cDNA, gRNAs targeting an exon-exon junction in each transgene (*TP53^R167H^* or *KRAS^G12D^*) were designed using CRISPOR web tool [36]. Each gRNA was synthesized by incubating equimolar ratios of Alt-R™ crRNA (Supplementary Table 3) and tracrRNA (#1072532; IDT Corporation, Chicago, IL, USA) at 95°C for 5 minutes and cooling to room temperature. Each gRNA was combined with purified *S. pyogenes* Cas9 nuclease (#1081058; IDT Corporation) to form a ribonucleoprotein (RNP) complex. Oncopig HCC cells were reverse transfected with 25 nM RNP complexes using the Lipofectamine CRISPRMAX kit (#CMAX00003; Invitrogen) following the manufacturer’s instructions.

### Confirmation of gene editing

Genomic DNA was extracted from Oncopig HCC cells using QuickExtract DNA Extraction Solution (#QE09050; Lucigen, Middleton WI, USA) following the manufacturer’s instructions. The genomic locus that flanks the Cas9 target site was amplified by PCR using primers listed in Supplementary Table 3. PCR products were provided to the Genome Editing Core at UIC, where a second PCR was performed to attach Fluidigm adaptor and barcode sequences. Targeted sequencing was performed using a MiSeq (Illumina, San Diego, CA, USA) following the manufacturer’s instructions. Sequencing reads were analyzed using CRISPResso2 with default parameters [37].

### Whole genome sequencing

DNA was extracted from Oncopig tissues and cell lines using the DNeasy Blood & Tissue Kit (Qiagen) following the manufacturer’s instructions. DNA (300 ng) was used to develop shotgun genomic libraries using the Hyper Library Construction Kit from Kapa Biosystems (Roche, Indianapolis, IN, USA) by the Roy J. Carver Biotechnology Center (University of Illinois, Urbana IL, USA) following standard protocols. Paired-end, 150 bp reads were generated by sequencing libraries on a NovaSeq 6000. All datasets are available in the NCBI Short Read Archive under accession number PRJNA599402.

### Intratumor heterogeneity

Sequencing reads were aligned to the Sscrofa11.1 reference genome using BWA MEM v0.7.17 [38]. Duplicate reads were marked using the GATK 4.1 MarkDuplicates function [39]. Following alignment, Strelka v2.9.10 [40] was used for somatic SNV calling using a multi-sample workaround described by Strelka’s developers (https://github.com/Illumina/strelka/issues/59), retaining variants flagged as PASS in at least one sample. HATCHet was used for somatic copy number alteration (CNA) calling [41]. Mutational signature exposure analysis was performed on the union of SNVs across all tumor samples using SignaturesEstimation [42]. To investigate the presence of SNVs in driver genes, homologous porcine genes were identified for a subset of 723 human driver genes from the COSMIC v90 database [43]. SNVs in driver genes were identified using bedtools v2.28.0 [44] and annotated using SnpEff [45].

### Statistical analysis

Comparison of Oncopig and human cell cycle length and gene expression was done using the Student’s *t*-test. Time to half gap closure, AFP secretion and cell proliferation at each time point were compared using 1-way analysis of variance (ANOVA). Pearson’s correlations were used to assess similarities of IC_50_ values across cell lines. Statistical analyses were performed using SPSS Statistics version 22 (SPSS Inc., Chicago IL, USA) and GraphPad Prism 8 (GraphPad, San Diego, CA, USA). *P*-values < 0.05 were considered statistically significant.

## SUPPLEMENTARY MATERIALS





## Abbreviations

HCC: hepatocellular carcinoma
LRT: locoregional therapy
UIC: University of Illinois at Chicago
RT-PCR: reverse transcription polymerase chain reaction
DMEM: Dulbecco’s Modified Eagle Medium
FBS: fetal bovine serum
CFSE: carboxyfluorescein succinimidyl ester
NIH: National Institutes of Health
5-FU: 5-fluorouracil
DMSO: dimethylsulfoxide
MTT: 3-(4,5-dimethylthiazol-2-yl)-2,5-diphenyltetrazolium bromide
IC_50_: half maximal inhibitory concentration
qPCR: quantitative polymerase chain reaction
RNA: ribonucleic acid
DNA: deoxyribonucleic acid
cDNA: complementary deoxyribonucleic acid
SCID: severe combined immunodeficiency
SQ: subcutaneous
CO_2_: carbon dioxide
PBS: phosphate buffered saline
AFP: alpha fetoprotein
CT: computed tomography
ELISA: enzyme-linked immunosorbent assay
IHC: immunohistochemistry
H&E: hematoxylin and eosin
CRISPR: clustered regularly interspaced short palindromic repeats
gRNA: guide ribonucleic acid
crRNA: CRISPR ribonucleic acid
tracrRNA: trans-activating CRISPR ribonucleic acid
RNP: ribonucleoprotein
bp: base pair
CNA: copy number alteration
SNV: single nucleotide variant
ANOVA: analysis of variance
KO: knockout
INDEL: insertion or deletion
AdCre: adenoviral vector encoding Cre recombinase
