# Supplementary material for: Development and comprehensive characterization of porcine hepatocellular carcinoma for translational liver cancer investigation
Source: Oncotarget. 2020 Jul 14;11(28):2686–701. doi: 10.18632/oncotarget.27647 (PMC7367657; doi:10.18632/oncotarget.27647)
Supplement: Supplementary file 1 [file oncotarget-11-2686-s001.pdf]

Development and comprehensive characterization of porcine hepatocellular carcinoma for translational liver cancer investigation

SUPPLEMENTARY MATERIALS

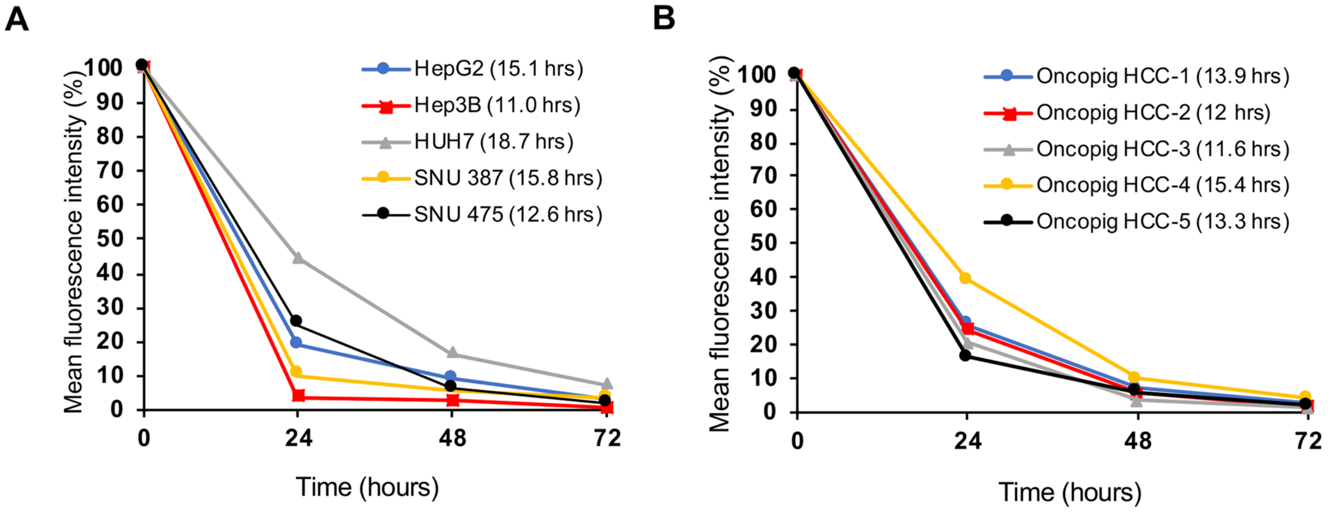

Supplementary Figure 1: Oncopig and human HCC cell cycle lengths. Cell cycle lengths for (**A**) five human and (**B**) five representative Oncopig HCC cell lines. Numbers in parentheses in the Figure legends represent average cell cycle lengths.

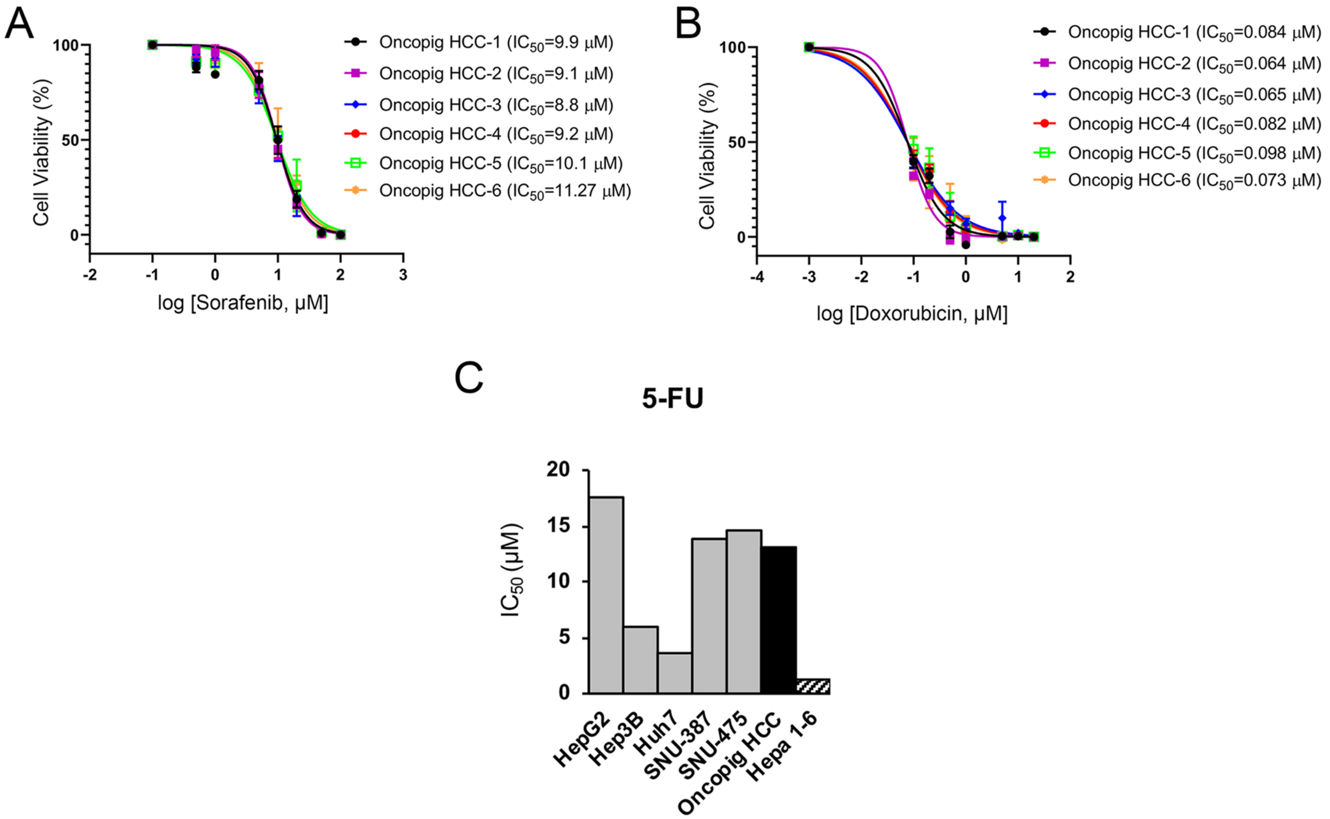

Supplementary Figure 2: Oncopig HCC *in vitro* chemotherapeutic susceptibility. (**A**) Sorafenib and (**B**) doxorubicin chemotherapeutic susceptibility of Oncopig HCC cell lines developed from distinct Oncopigs (n = 6). (**C**) IC_{50} values demonstrating increased susceptibility of murine (Hepa1-6) compared to Oncopig and human HCC for 5-FU.

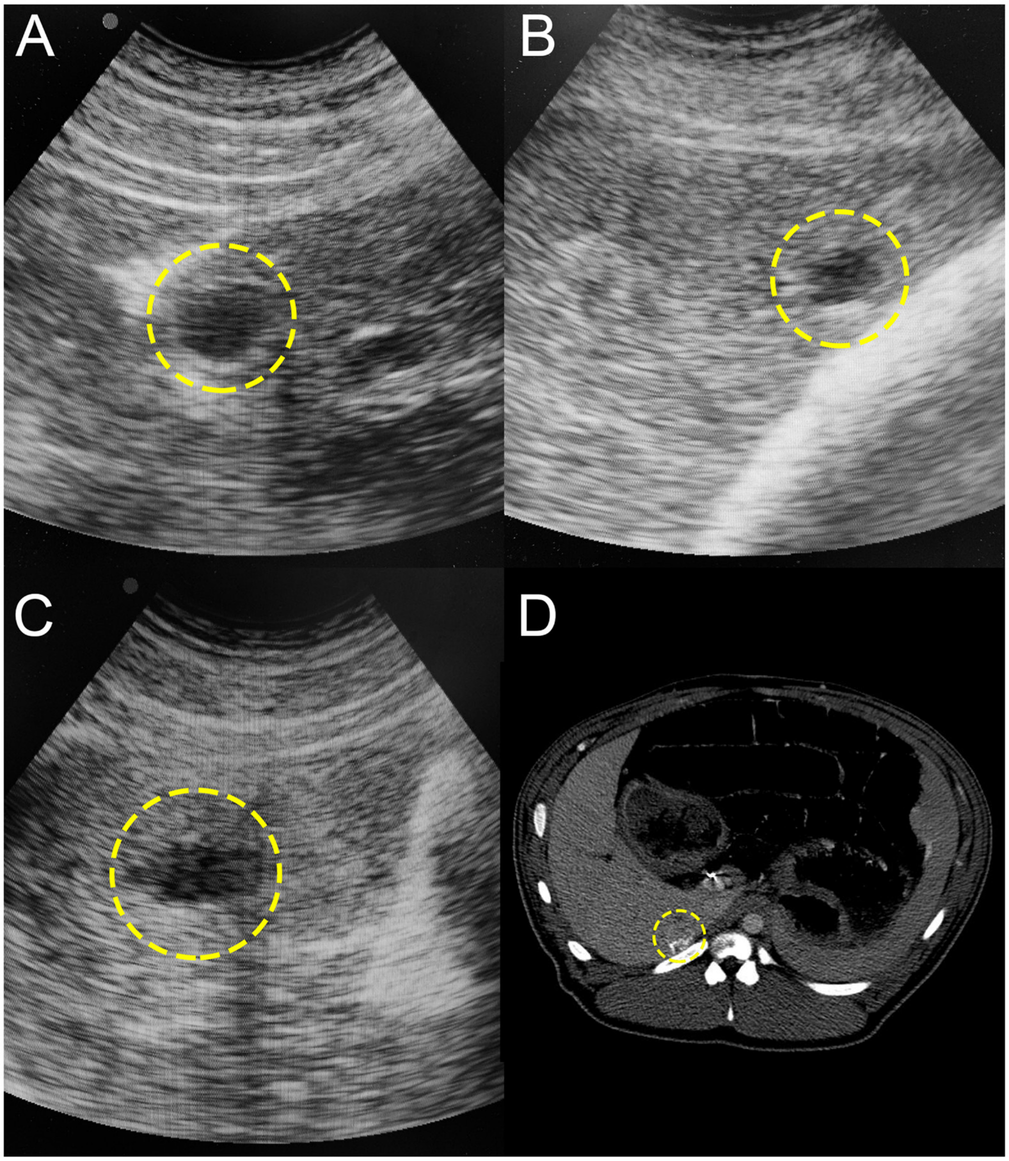

Supplementary Figure 3: Reproducible generation of Oncopig intrahepatic HCC tumors. (**A**) 4-week post engraftment liver ultrasound depicting a 1.0 cm hypoechoic intrahepatic mass in one Oncopig that was not visualized at later time points. Liver ultrasound demonstrating (**B**) 0.6 cm hypoechoic intrahepatic HCC 2-weeks post engraftment that increased to (**C**) 1.4 cm in size at 4-weeks post engraftment. (**D**) Contrast enhanced liver CT 10-weeks post engraftment depicts 1.0 cm HCC tumor (circled) depicted in (B) and (C).

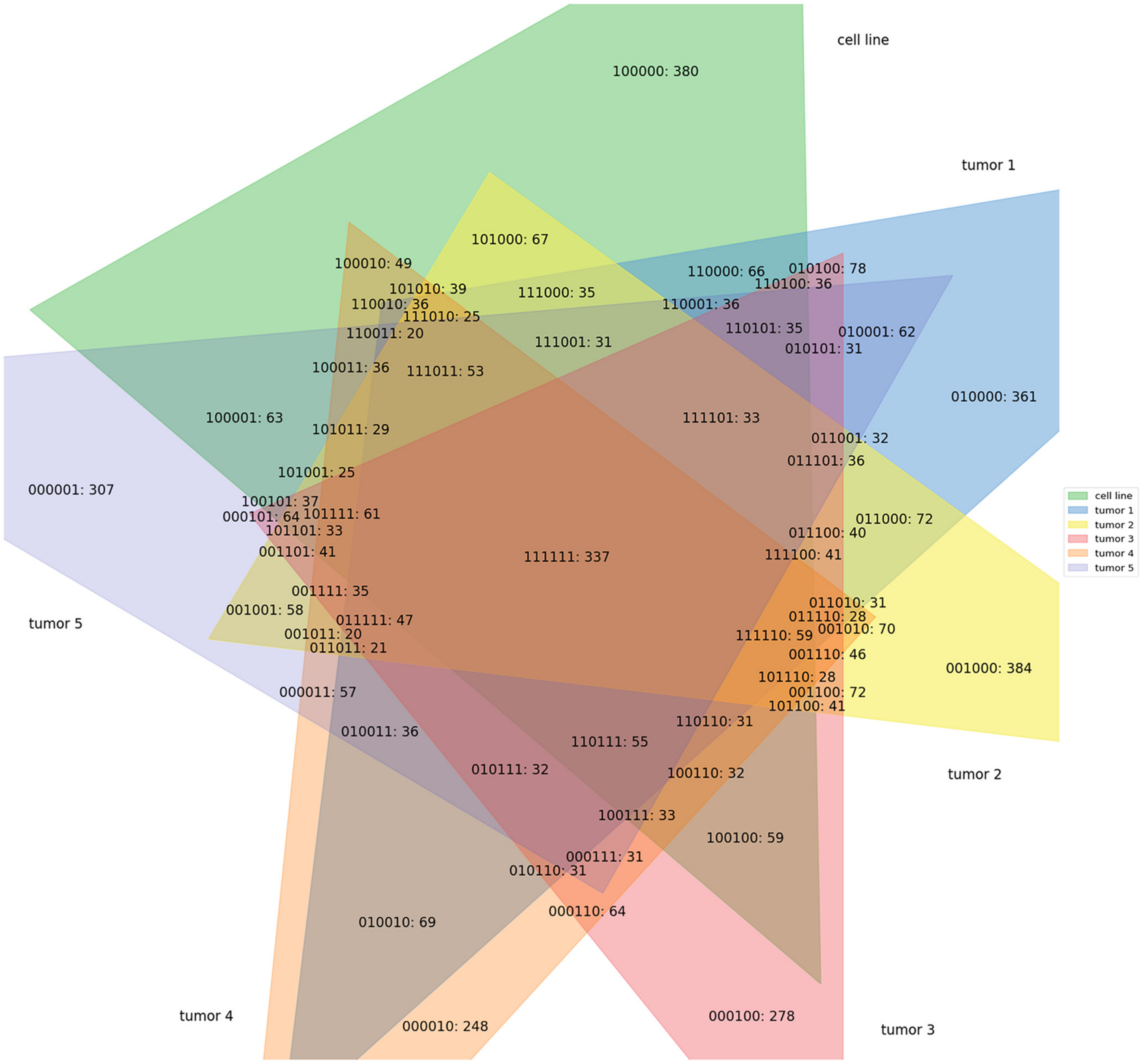

Supplementary Figure 4: Distribution of SNVs across Oncopig intrahepatic tumor biopsies. Venn diagram depicting unique and shared SNVs detected in the 5 intrahepatic HCC tumor biopsies and the HCC cell line used to generate the tumor.

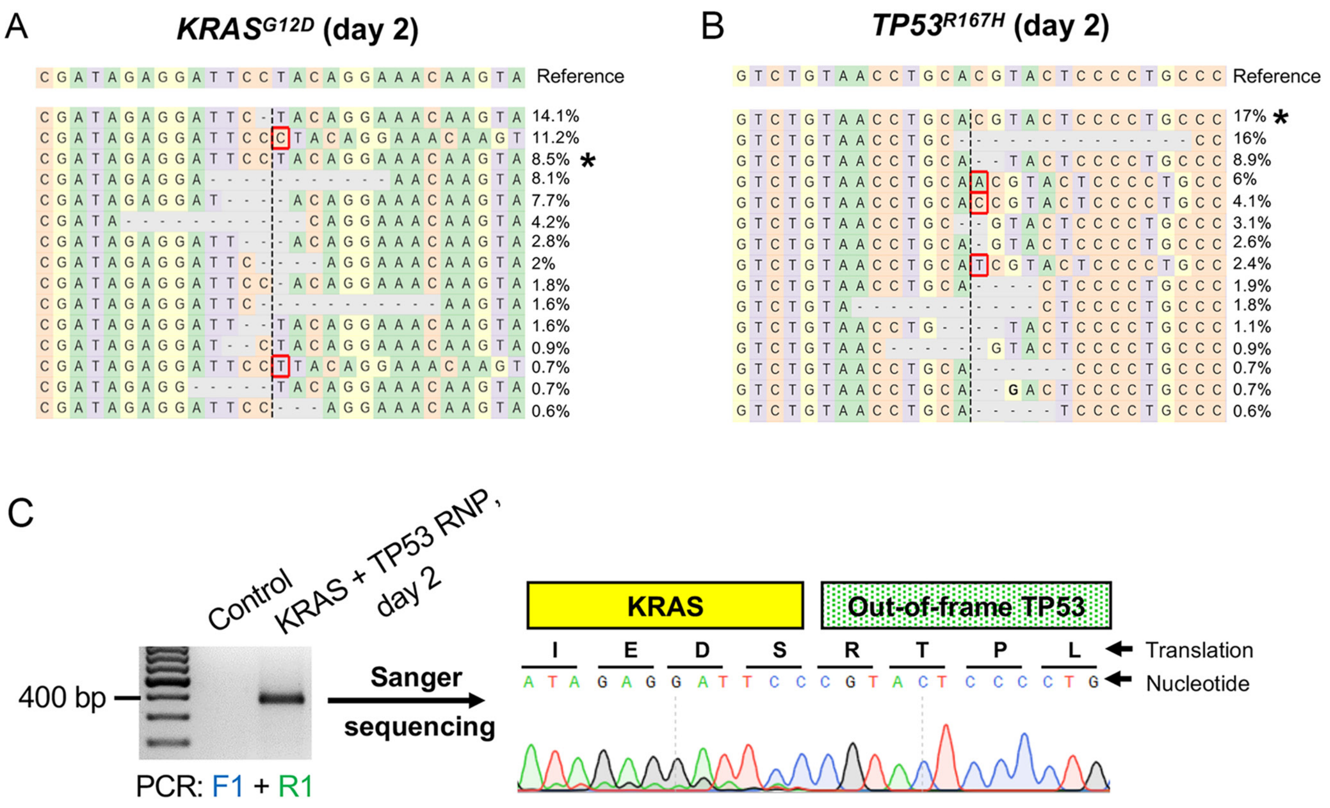

Supplementary Figure 5: Oncopig HCC cell line gene editing results. Most frequent INDELs detected by targeted Illumina sequencing 2 days post transfection for Oncopig HCC cells transfected with gRNA targeting (**A**) *KRAS*^{G12D} or (**B**) *TP53*^{R167H}. Asterisks indicate unedited reads. Dashed line, cleavage position; red box, insertion; dashed grey box, deletion. (**C**) Sequence analysis for Oncopig HCC cells co-transfected with gRNAs targeting *KRAS*^{G12D} and *TP53*^{R167H}, confirming deletion of the region between the *KRAS*^{G12D} and *TP53*^{R167H} transgenes.

Supplementary Table 1: Chemotherapeutic response of liver cancer cell lines

Sorafenib	IC_{50} (μM)	$\log IC_{50}$ ± S. E.
HepG2	7.6	0.878 ± 0.048
Hep3B	3.5	0.545 ± 0.204*
Huh7	2.0	0.308 ± 0.016*#
SNU-387	14.7	1.168 ± 0.051#
SNU-475	16.2	1.211 ± 0.052#
Oncopig HCC	9.9	0.995 ± 0.032
Hepa1-6	5.6	0.750 ± 0.024
**Doxorubicin**	**IC_{50}**	**$\log IC_{50}$ ± S. E.**
HepG2	0.26	–0.579 ± 0.023*
Hep3B	0.43	–0.365 ± 0.025*
Huh7	0.14	–0.847 ± 0.042#
SNU-387	0.89	–0.050 ± 0.029*
SNU-475	3.98	0.600 ± 0.225*#
Oncopig HCC	0.084	–1.076 ± 0.049#
Hepa1-6	0.51	–0.292 ± 0.023*
**Cisplatin**	**IC_{50}**	**$\log IC_{50}$ ± S. E.**
HepG2	7.2	0.855 ± 0.064#
Hep3B	4.9	0.686 ± 0.042#
Huh7	1.8	0.252 ± 0.055*
SNU-387	27.7	1.442 ± 0.052*#
SNU-475	17.7	1.247 ± 0.058*#
Oncopig HCC	5.7	0.754 ± 0.024#
Hepa1-6	2.2	0.334 ± 0.029*
**Mitomycin C**	**IC_{50}**	**$\log IC_{50}$ ± S. E.**
HepG2	0.60	–0.219 ± 0.037
Hep3B	5.25	0.720 ± 0.072*#
Huh7	0.65	–0.188 ± 0.060
SNU-387	4.30	0.634 ± 0.107*#
SNU-475	1.72	0.234 ± 0.043*#
Oncopig HCC	0.68	–0.165 ± 0.049
Hepa1-6	0.68	–0.164 ± 0.038
**5-FU**	**IC_{50}**	**$\log IC_{50}$ ± S. E.**
HepG2	17.6	1.245 ± 0.063#
Hep3B	6.0	0.776 ± 0.147#
Huh7	3.6	0.559 ± 0.101*#
SNU-387	13.9	1.142 ± 0.100#
SNU-475	14.6	1.165 ± 0.071#
Oncopig HCC	13.1	1.117 ± 0.082#
Hepa1-6	1.3	0.097 ± 0.117*

Supplementary Table 2: Driver mutations identified in intrahepatic Oncopig HCC tumor biopsies.
See Supplementary Table 2

Supplementary Table 3: Oligonucleotides used in the study

**RT-PCR Primers**	
KRAS^{G12D}	ttgtacagctagctgctgaaaatgactgaatat, attctcgagcggttacataattatacac
TP53^{R167H}	tggctctcctcaagcgtatt, attttcatccagccagttcg
**qPCR Primers**	
**Pig genes**	
SLC22A1	ctcgaagaggaggtcgtcac, cggtcgatgatgaggagaat
CYP3A39	ggctaccgtaagggtgttga, tcctcagccagagagagagc
UGT1A1	ctgctcccacttactgcaca, ggcaatccattcaggaagaa
CBR1	acaggaaggagggatggagt, gctcctacctctgggctctt
ABCB1	atggcagtgggacaggttag, ggtcgagtggggtagttgaa
GAPDH	tcatcatctctgccccttct, gtcatgagtccctccacgat
**Human genes**	
SLC22A1	taatggaccacatcgctcaa, agcccctgatagagcacaga
CYP3A4	caagacccctttgtggagaa, cgaggcgactttctttcatc
UGT1A1	tgcagatggttgcaattgat, cattctttttccccaaagca
CBR1	atacggggtgacgaagattg, caccttcttctgggctcttg
ABCB1	gctcctgactatgccaaagc, tcttcacctccaggctcagt
GAPDH	gagtcaacggatttggtcgt, ttgattttggagggatctcg
**crRNAs**	
KRAS^{G12D}	ctacgatagaggattcctac
TP53^{R167H}	gagggcaggggagtacgtgc
**Targeted Sequencing Primers**	
KRAS^{G12D}	agttggagctgatggcgtag, aatggtgaatatcttcaaatgactt
TP53^{R167H}	catatgcaatggaggagtcg, ccagctggcaaaacagctta
**PCR Primers**	
**F1**	gctagctgctgaaaatgactga
**R1**	ccaagtactcggcccgtaaa
**R2**	attctcgagcggttacataattatacac
